# Biological Mesh Repair of a Large Incisional Hernia Containing a Kidney Transplant in the Presence of Inflammation

**DOI:** 10.1155/2020/5675613

**Published:** 2020-03-24

**Authors:** Harkiran Sran, Miriam Manook, Pankaj Chandak, Raphael Uwechue, Martin Drage, Ioannis Loukopoulos, Nicos Kessaris

**Affiliations:** Department of Nephrology and Renal Transplantation, Guy's Hospital, London, UK

## Abstract

The incidence of incisional hernia after kidney transplantation varies between 1.1% and 3.8%. These are usually repaired electively using polypropylene mesh. We present here a case where a patient presented as an emergency, with a large painful incisional hernia over his kidney transplant, and evidence of local erythema and systemic inflammation. As this could have represented either infection or rejection, the patient was started on antibiotics and subsequently underwent graft nephrectomy and hernia repair using a biological (porcine-derived) acellular dermal matrix, Strattice™, with a satisfactory outcome. In addition, histology showed evidence of allograft rejection. This is the first reported case of an incisional hernia containing a rejecting kidney allograft, managed with nephrectomy and biological mesh repair.

## 1. Introduction

The reported incidence of incisional hernia after kidney transplantation is lower than that following abdominal surgery (up to 20%) [1]. This may be due to the fact that the incision is usually a “hockey-stick” or modified Rutherford Morrison incision on the right or left side of the abdominal wall. Risk factors for hernia include obesity, infection, reoperation, and immunosuppression [1]. Such hernias are usually repaired electively using polypropylene mesh, but other methods include primary fascial approximation and the use of autologous tensor fascia lata graft [1–3]. The latter method has been used in the past when there is tissue deficiency in the presence of contamination. More recently, biological meshes have been used widely in contaminated abdominal wounds, but to a much smaller extent following transplantation. This case discusses the use of one such mesh in a case where infection could not be excluded.

## 2. Case Presentation

A 52-year-old nondiabetic male, with a history of tubulointerstitial nephritis, underwent a routine extraperitoneal renal transplant to the right iliac fossa from a 59-year-old DCD (donor after circulatory death). The HLA mismatch was 1 : 2 : 0, and the cold ischaemic time was 10 hours. Induction immunosuppression was with Simulect (basiliximab) at the time of transplantation and on day 4, as well as methylprednisolone. Subsequent maintenance immunosuppression was with tacrolimus (Adoport), mycophenolate mofetil, and prednisolone. His first deceased donor kidney transplant (in the left iliac fossa) had lasted 10 years before it was removed for vascular rejection two years after graft failure and return to dialysis.

**Figure 1:**
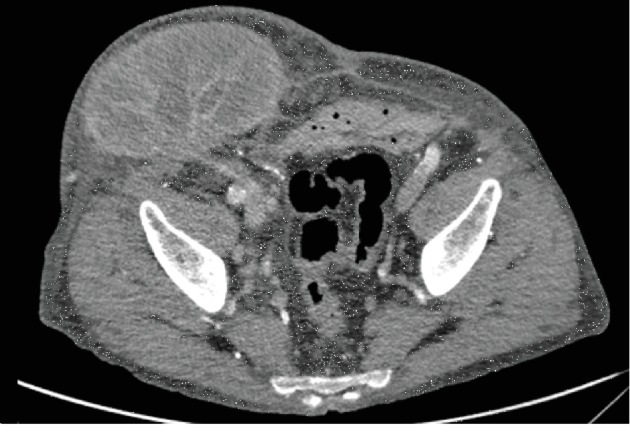
CT scan demonstrating the incisional hernia containing the transplant kidney on the right side of the abdominal wall.

**Figure 2:**
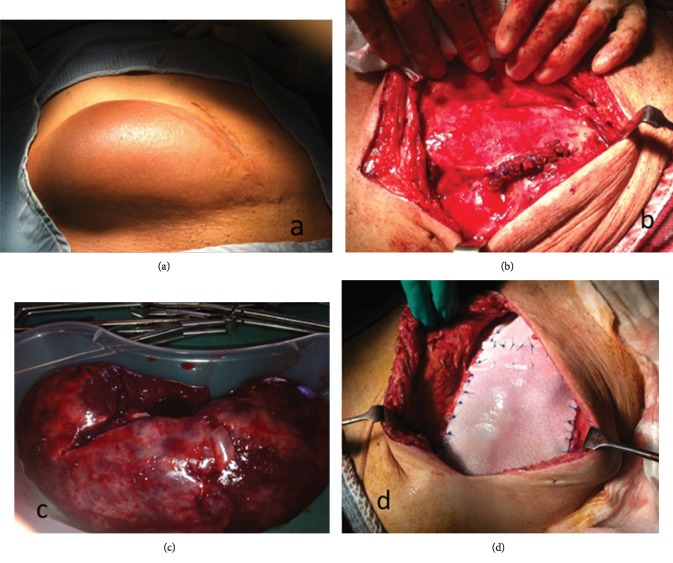
(a) demonstrates the incisional hernia containing the transplant kidney before surgery. (b) shows the partly infarcted kidney after transplant nephrectomy. (c) displays the hilum and vessels secured with 2/0 and 5/0 prolene sutures. (d) illustrates the reconstruction of the abdominal wall defect using biological porcine skin mesh (Strattice™).

Initial delayed graft function was attributed to early vascular (T-cell) and humoral (antibody mediated) rejection, which was treated with additional immunosuppression (Thymoglobulin). Despite improvement in renal function, the patient then developed BK nephropathy necessitating reduction in immunosuppression. Unfortunately, despite treating a further episode of rejection, renal function did not improve, and he returned to haemodialysis eight months after transplantation. Maintenance immunosuppression with tacrolimus was stopped at this point, but low-dose prednisolone was continued. Three months later, the patient was admitted with a mass in the right iliac fossa that had progressively increased in size. CT scan showed the transplant kidney to be 12.7 cm and lying very superficially (Figure 1). It demonstrated poor enhancement in keeping with the appearance of infarction. He was given a course of cephalexin and a date a few days later for transplant nephrectomy. At the time of admission, the patient was pyrexial at 39°C, there was evidence of local erythema and systemic inflammation (Figure 2(a)), and CRP was elevated at 188 mg/L. As an infective cause could not be excluded, intravenous coamoxiclav was commenced prior to surgery.

Operative findings revealed a grossly enlarged kidney contained within the hernia sac and a large abdominal wall defect. First, open nephrectomy was performed (Figure 2(b)) by securing the hilum and vessels with 2/0 and 5/0 prolene sutures, respectively (Figure 2(c)). Reconstruction of the abdominal wall defect was then performed using two pieces (8 cm × 16 cm each) of Strattice™ porcine-derived acellular dermal matrix sutured together with an onlay placement. The mesh overlapped surrounding healthy tissue by 3-4 cm and was fixed with interrupted 2/0 prolene sutures over the inguinal ligament inferiorly, the rectus sheath superiorly and medially, and the external oblique aponeurosis and muscle superiorly and laterally (Figure 2(d)).

Postoperatively, the patient was continued on intravenous antibiotics appropriate to culture findings as per advice from microbiologists. His clinical condition improved over a few days with resolution of all signs of inflammation. Two subsequent blood cultures demonstrated no growth. After a total inpatient stay of 10 days, the patient was discharged home. Histology of the kidney transplant showed evidence of acute and chronic vascular and humoral rejection with renal cortical necrosis. Apart from slight muscle weakness, there was no fascial defect, and no clinical nor radiological evidence of recurrence at last follow-up several years later.

## 3. Discussion

Synthetic mesh is commonly used to repair incisional hernias in the elective setting [1]. Various biological meshes have also been used in the management of herniae following kidney, liver, and multivisceral transplants [2, 3] but rarely in the presence of inflammation or infection. The Ventral Hernia Working Group expert consensus does not recommend the use of synthetic mesh in the presence of infection, instead promoting the use of a bioprosthetic mesh [4].

In nontransplant abdominal wall hernias, where there is a high risk of surgical site infection, the use of autologous fascia grafts and biological meshes has been previously described [4–6]. Types of biological meshes include AlloDerm®, Permacol™, and Surgisis™ [6, 7]. Smart et al. [6] describe Permacol™ to have the lowest failure rate and the longest time to failure in contaminated areas, likely associated with the fact that it is a cross-linked porcine mesh and more durable [4].

Coccolini et al. [8] describe 70 cases of abdominal wall hernias following transplantation, including kidney, liver, pancreas, bowel, and multivisceral transplants. Porcine dermal collagen was used in 24.3% of these cases, human dermal collagen in 51.4%, and swine intestinal submucosa in 24.3%. The average complication rate was 9.4%, and the group concluded that the biological meshes were useful and safe in repairing abdominal wall hernias in transplanted patients.

Strattice™ allows cell repopulation, revascularization, and migration of white cells thus allowing resistance to infection. This is a non-cross-linked matrix, but Cheng et al. [9] reported an association with fewer short-term complications in 75 cases when compared with 195 abdominal wall hernias repaired with Permacol™. The hernia recurrence rate was similar when comparing the two.

In summary, to our knowledge, this is the first reported case of a large incisional hernia containing a rejected transplant kidney repaired with the use of a biological matrix. This was in the presence of inflammation while awaiting blood cultures. These did not show any eventual evidence of infection, but our patient had already been on antibiotics before these were performed. This case demonstrates that the use of biological mesh is feasible and effective for repairing large incisional hernias in selected renal transplant recipients, even in the presence of significant inflammation.
